# Tissue catabolism and donor-specific dexamethasone response in a human osteochondral model of post-traumatic osteoarthritis

**DOI:** 10.1186/s13075-022-02828-4

**Published:** 2022-06-10

**Authors:** Rebecca Mae Black, Lisa L. Flaman, Karin Lindblom, Susan Chubinskaya, Alan J. Grodzinsky, Patrik Önnerfjord

**Affiliations:** 1 Department of Biological Engineering, Massachusetts Institute of Technology, Cambridge, MA USA; 2 Rheumatology and Molecular Skeletal Biology, Department of Clinical Sciences Lund, Faculty of Medicine, Lund University, Lund, Sweden; 3 Departments of Pediatrics, Orthopedic Surgery and Medicine (Section of Rheumatology), Rush University Medical Center, Chicago, IL USA; 4 Department of Mechanical Engineering, Massachusetts Institute of Technology, Cambridge, MA USA; 5 Department of Electrical Engineering and Computer Science, Massachusetts Institute of Technology, Cambridge, MA USA

**Keywords:** Post-traumatic osteoarthritis, Mass spectrometry, Cartilage matrix, Cytokines, Proteomics, Dexamethasone, Biomarkers

## Abstract

**Background:**

Post-traumatic osteoarthritis (PTOA) does not currently have clinical prognostic biomarkers or disease-modifying drugs, though promising candidates such as dexamethasone (Dex) exist. Many challenges in studying and treating this disease stem from tissue interactions that complicate understanding of drug effects. We present an ex vivo human osteochondral model of PTOA to investigate disease effects on cartilage and bone homeostasis and discover biomarkers for disease progression and drug efficacy.

**Methods:**

Human osteochondral explants were harvested from normal (Collins grade 0–1) ankle talocrural joints of human donors (2 female, 5 male, ages 23–70). After pre-equilibration, osteochondral explants were treated with a single-impact mechanical injury and TNF-α, IL-6, and sIL-6R ± 100 nM Dex for 21 days and media collected every 2–3 days. Chondrocyte viability, tissue DNA content, and glycosaminoglycan (sGAG) percent loss to the media were assayed and compared to untreated controls using a linear mixed effects model. Mass spectrometry analysis was performed for both cartilage tissue and pooled culture medium, and the statistical significance of protein abundance changes was determined with the R package limma and empirical Bayes statistics. Partial least squares regression analyses of sGAG loss and Dex attenuation of sGAG loss against proteomic data were performed.

**Results:**

Injury and cytokine treatment caused an increase in the release of matrix components, proteases, pro-inflammatory factors, and intracellular proteins, while tissue lost intracellular metabolic proteins, which was mitigated with the addition of Dex. Dex maintained chondrocyte viability and reduced sGAG loss caused by injury and cytokine treatment by 2/3 overall, with donor-specific differences in the sGAG attenuation effect. Biomarkers of bone metabolism had mixed effects, and collagen II synthesis was suppressed with both disease and Dex treatment by 2- to 5-fold. Semitryptic peptides associated with increased sGAG loss were identified. Pro-inflammatory humoral proteins and apolipoproteins were associated with lower Dex responses.

**Conclusions:**

Catabolic effects on cartilage tissue caused by injury and cytokine treatment were reduced with the addition of Dex in this osteochondral PTOA model. This study presents potential peptide biomarkers of early PTOA progression and Dex efficacy that can help identify and treat patients at risk of PTOA.

**Supplementary Information:**

The online version contains supplementary material available at 10.1186/s13075-022-02828-4.

## Introduction

Though millions of patients worldwide suffer from osteoarthritis (OA), no disease-modifying OA drug (DMOAD) has been approved due to many challenges in the drug development pipeline, and promising candidates often fail at the level of clinical trials [1–3]. Post-traumatic OA (PTOA) is an important disease target since the time of the disease onset (the injury) is known. Many in vitro models of PTOA using human cartilage and isolated chondrocytes offer insights into disease progression, but do not capture the complexity of a full joint that has vasculature, bone, and synovial cross-talk [4]. Models of OA progression using osteochondral explants exist, but have used either diseased knee arthroplasty discards or stem cell-derived engineered organoids that do not completely recapitulate an in vivo healthy control state [5–8]. Therefore, it is desirable to use a model starting with healthy primary human cartilage and bone to better understand disease progression and drug effects on healthy as well as diseased joint tissues.

Outcome measures of drug efficacy often focus on late-stage pain or structural changes, while early tissue breakdown events can progress years before macroscopic changes are observed. Molecular biomarkers of disease progression can offer insight into early disease processes that could help in drug development as therapeutic endpoints, as well as potential prognostic measurements of patients for the earliest stages of PTOA. In a previous model of PTOA using human knee cartilage explants, mass spectrometry analysis of culture media identified the time-dependent release of proteases and catabolic signaling processes within days after mechanical injury and inflammatory cytokine exposure, hypothesized to be potential biomarkers of disease progression [9]. However, this in vitro model did not incorporate tissue crosstalk that could affect signaling processes and only focused on changes in the media proteome and not the cartilage tissue. Another set of potential biomarker targets is protein fragments produced by enzymatic activity not present in healthy cartilage. A neoepitope of cartilage oligomeric protein (COMP) produced by proteolytic activity was discovered in the synovial fluid of patients and later validated in a human knee cartilage explant model of PTOA, and there is potential to identify fragments of other proteins that are generated under disease stress [10, 11].

In the search for DMOADs for patients at risk of PTOA development, corticosteroids have been identified as promising therapeutics. One of the most well-studied is dexamethasone (Dex), the most potent of the corticosteroid family, currently used for its analgesic effects [12]. In human and animal cartilage tissue models of PTOA, Dex exerted anti-catabolic effects on matrix breakdown and protease production [9, 13–15]. However, there are concerns over the safety of Dex use, as in some models using isolated chondrocytes, higher doses of Dex have been shown to cause chondrocyte death [16, 17]. In models using cartilage explants, negative effects on cartilage viability are not commonly observed; however, Dex can exacerbate changes in immune signaling late into disease treatment [9, 14]. Long-term systemic corticosteroid treatment has also been shown to increase incidence of osteoporosis, leading to concerns over effects on bone homeostasis [18]. It is of interest to identify which patients might be the most or least responsive to Dex to target treatment, as there is variability in the response to both disease and Dex at the cellular level [19, 20].

In the present study, we use a human ankle osteochondral model of PTOA, starting with initially normal donor joints, to study early disease progression and to better understand catabolic and anabolic responses in the context of both cartilage and bone. Ankle OA is estimated to have an ~8% prevalence, rarer than hip or knee OA but non-negligible, and up to 90% of the incidence of ankle OA is post-traumatic [21, 22]. This discovery-mode analysis of both the cartilage and media proteomes allows identification of potential biomarkers of PTOA progression and proteolytic events and analysis of donor-specific responses to Dex. We hypothesize that, in this model of PTOA that incorporates cartilage-bone crosstalk, (1) the cartilage and bone tissues will undergo catabolic degradation, (2) anabolic and homeostatic cellular processes will differ from control, and (3) donors will respond differently to disease and drug treatment due to donor-specific differences in cartilage and bone biology.

## Materials and methods

### Explant harvest and treatment

**Fig. 1:**
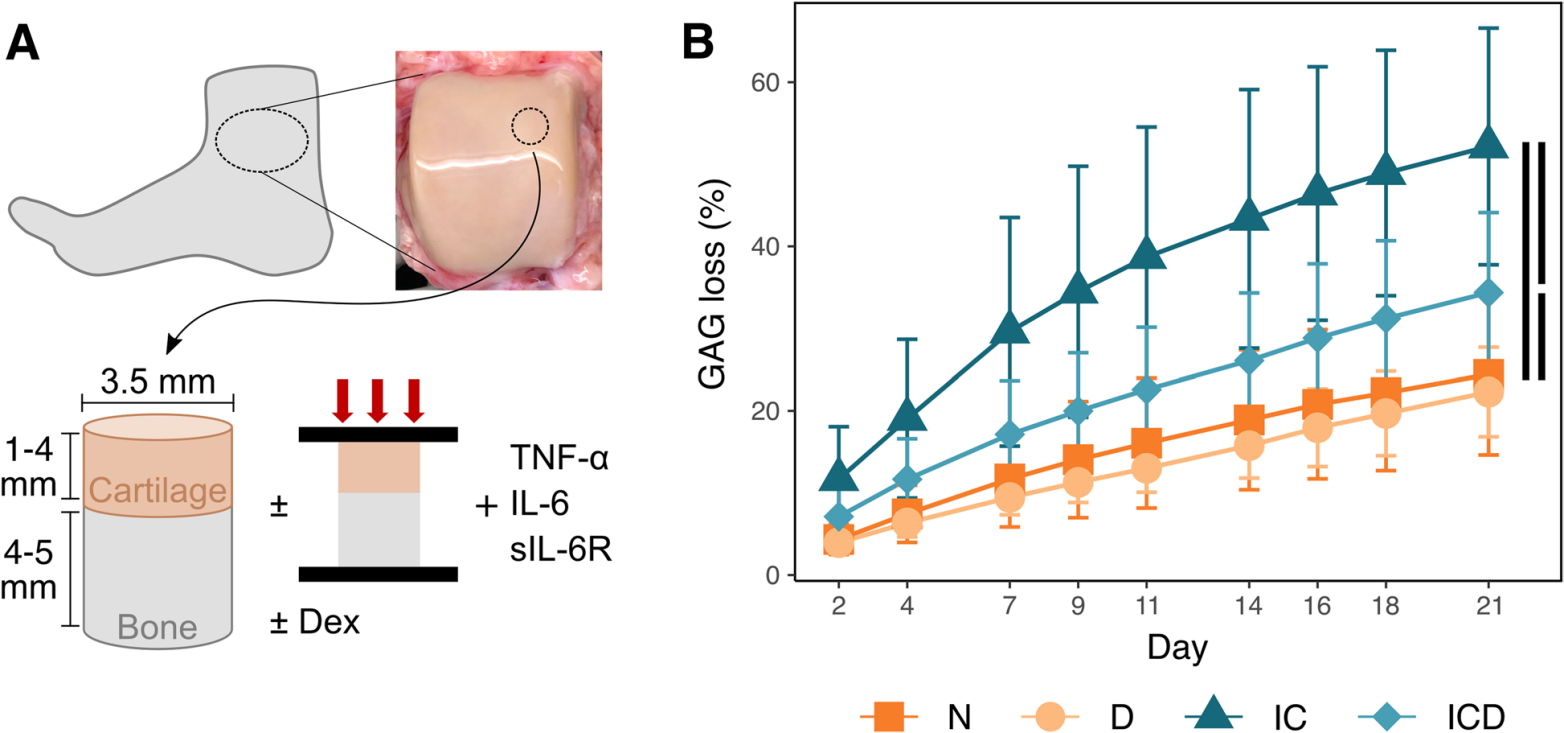
Methods overview and percent sGAG loss across all donors. **A**. Grade 0–1 osteochondral plugs containing full-thickness cartilage and 4-5 mm of the underlying bone were harvested from the talus joints of seven adult human donors. Plugs were left untreated, treated with 100 nM Dex, subjected to a single impact mechanical injury and cultured with 25 ng/mL TNF-α, 50 ng/mL IL-6, and 250 ng/mL sIL-6R, or treated with injury, cytokines, and Dex for 3 weeks. **B**. Summed total percent sGAG loss from the tissue to the medium over 3 weeks across all donors. N, no treatment; D, Dex alone treatment; IC, injury and cytokine treatment; ICD, injury, cytokines, and Dex. Error bars represent standard deviation. Bar: *p* < 0.05, day 21

Human osteochondral explants (3.5 mm diameter, full-thickness cartilage, and ~4 mm bone) were harvested from ankle talocrural joints of seven human donors (62F, 66M, 66M, 44F, 23M, 39M, 70M, Collins grade 0–1) obtained postmortem within 48 h of death through the Gift of Hope Organ and Tissue Donor Network (Itasca, IL). Explants were pre-equilibrated for two days in high glucose phenol red-free Dulbecco’s Modified Eagle Medium (DMEM) (Thermo Fisher) before switching to low-glucose phenol red-free DMEM, supplemented as described [15]. After pre-equilibration, osteochondral explants were treated for 21 days ± a single-impact mechanical injury (60% final strain at 300%/s strain rate; both followed by immediate release at the same rate [11, 23]) and inflammatory cytokines (25 ng/mL tumor necrosis factor alpha (TNF-α) + 50 ng/mL interleukin-6 (IL-6) + 250 ng/mL soluble IL-6 receptor (sIL-6R); treatment IC), as well as with 100 nM Dex alone (D) or treatment IC plus 100 nM Dex (ICD) (Fig. 1A). The use of these cytokines to simulate early inflammation in PTOA has been well-established in previous studies [13, 24]. All donors provided both left and right ankles, and explants from one ankle were used for proteomic analysis while the other ankle was used for sGAG and DNA biochemical analysis. Viability analysis was performed on samples randomized from both ankles. Culture medium was collected and stored at −20°C until analysis.

### Biochemical and viability analysis

Cartilage tissue was removed from the underlying bone and digested in 1 mg/mL proteinase K (Sigma). Percent loss of sGAG content from digested cartilage tissue into the medium was determined using the dimethylmethylene blue (DMMB) assay [25], and tissue DNA content was quantified with the Quanti-iT PicoGreen dsDNA kit (Thermo) according to manufacturer instructions. Significance for biochemical measurements was determined fitting the data to a linear mixed effects model with donor as a random effect followed by a least squares means test, using the R package lmerTest. To determine chondrocyte viability within the cartilage, 100–200 μm vertical slices were cut from intact cartilage and stained with fluorescein diacetate and propidium iodide (Sigma) as previously described [14].

### Mass spectrometry preparation and identification

Culture medium (50 μL) was prepared for mass spectrometry (MS) analysis as described [11, 15]. Cartilage tissue samples were removed from the underlying bone and prepared for MS analysis as described [26]. Discovery MS was performed using a quadrupole Orbitrap benchtop mass spectrometer (Q-Exactive HFX, Thermo Scientific) with prior separation of peptides using a liquid chromatography system (EASY-nLC 1000, Thermo Scientific) on an analytical column (PepMap RSLC C18, 75μm × 25cm, Thermo Scientific) coupled on-line using a nano-electrospray ion source with a column temperature at +45°C (EASY-Spray, Thermo Scientific) using a flow rate of 300nL/min. Protein identification was performed in Proteome Discoverer 2.5 (Thermo Scientific) using two search engines in parallel: a tryptic search against the UniProt human (UP000005640 from 2021-01) sequence database combined with an MSPep spectral search against the NIST_human_Orbitrap_HCD_20160923 library (mass tolerance: 10 and 20 ppm in MS1, MS2 respectively). Other Sequest search settings were modifications: carbamidomethylation (fixed: C), oxidation (variable: M, P) missed cleavages (max 2), mass tolerance (MS1-10ppm, MS2-0.02Da). Label-free protein abundance quantification was obtained by averaging peak area intensities from the top three unique peptides for each protein. To determine individual peptide abundances, we performed a semi-tryptic database search to enable identification of non-tryptic cleavages within the dataset. This was performed using the same combined searches as above but in series. The protein false discovery rate (FDR) was 0.01 for both searches.

### Bioinformatics analyses

For both the peptide and protein MS data sets, proteins were filtered out if they were exogenous or not identified and quantified in at least 70% of samples, and missing values were imputed using the *k*-nearest neighbor method [9, 27]. Cartilage tissue data was normalized to the DNA content per wet weight of cartilage tissue to adjust for different cell densities between donors (Supplemental Figure 1A). The media data had significant batch effects due to two collections of donors (Supplemental Figure 1B), so data used for principle component and regression analyses were batch corrected using the R limma package function “removeBatchEffect” [28]. Protein and peptide abundance data were log_2_-transformed and scaled, and principle component analysis (PCA) was performed using the “prcomp” function [15]. Pairwise comparisons between treatments were performed on the individual peptide and protein abundances. Statistical analysis on proteomic and peptide data was performed using limma and MATLAB (MathWorks). Protein and peptide abundances were regressed using partial least squares regression against total sGAG loss or the percent of sGAG loss that was attenuated by Dex treatment, which yielded a dot product of the first two loading vectors for each protein or peptide. The proteins and peptides were ranked by their dot product and analyzed using Gene Set Enrichment Analysis (GSEA) c5 gene sets with the Human UniProt IDs chip and 1,000 repeats for enrichment score distributions [29, 30]. Enrichment analysis for biological processes was performed using the PANTHER database and STRING analysis as previously described [15, 31].

## Results

### Cartilage viability and sGAG loss during disease and drug treatment

Osteochondral plugs from seven pairs of Collins grade 0-1 human donor ankles were successfully cultured for 3 weeks. Without any treatment, the cartilage tissue lost around 20% sGAG content and maintained chondrocyte viability, consistent with other studies using isolated cartilage (Fig. 1B, Supplemental Figure 2). Low-dose treatment of only Dex had no effect on cell viability or sGAG loss, while treatment with injury and cytokines (IC) caused widespread cell death in the cartilage and increased sGAG loss significantly, up to 54%. The addition of Dex to IC treatment (ICD) ameliorated sGAG loss by 2/3, though not back to control levels, and reduced the amount of chondrocyte death. There were donor-specific differences in how much sGAG loss each individual donor experienced, as well as the degree to which Dex attenuated sGAG loss, if at all (Supplemental Figure 3).

### Mass spectrometry identification of proteins in media and cartilage tissue and disease and Dex effects on media and cartilage proteomes

**Fig. 2:**
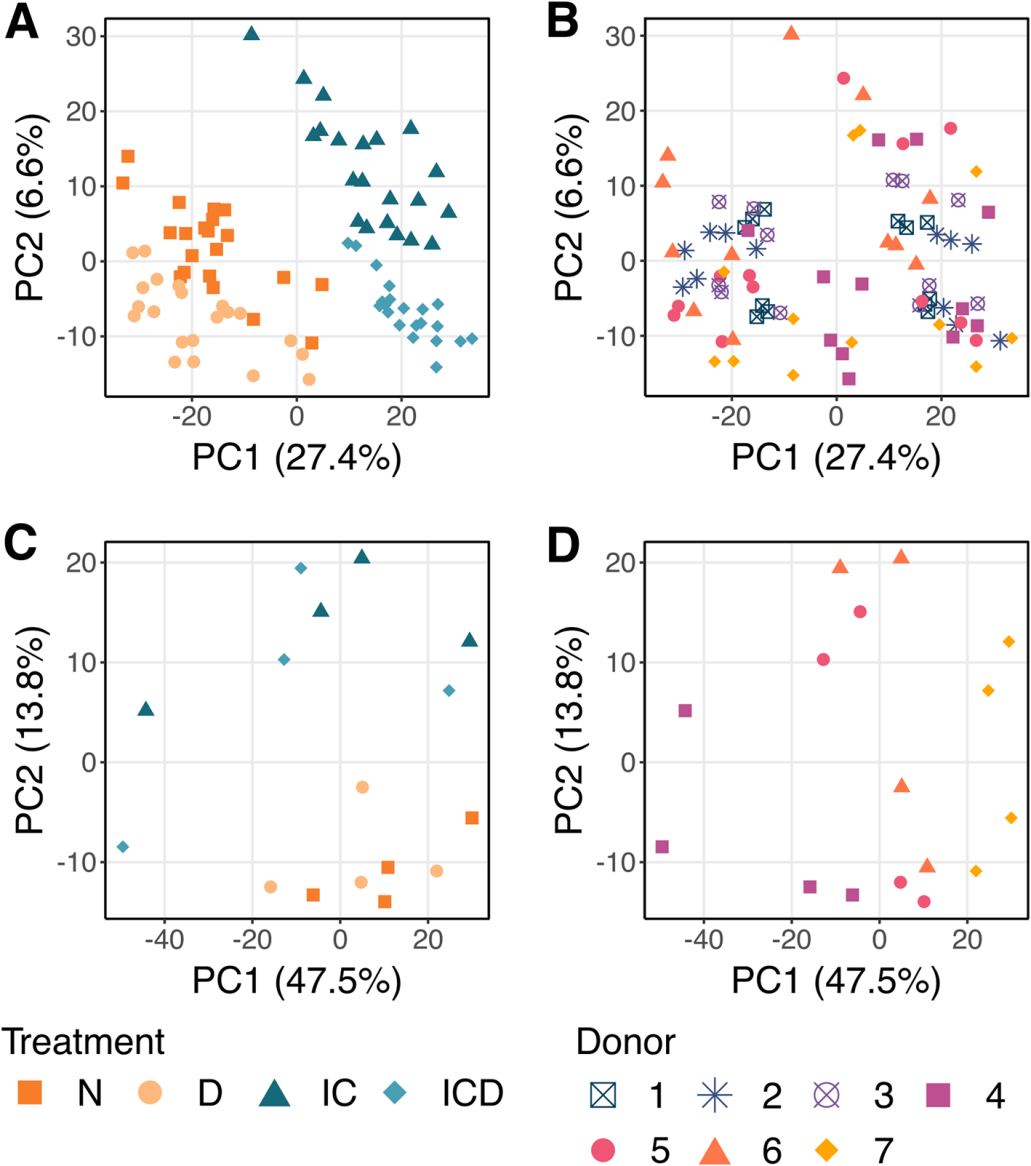
Principal component analysis of media and tissue proteomes. PCA was performed on filtered and imputed proteomic data for batch-corrected media samples (**A**, **B**) and DNA-normalized tissue samples (**C**, **D**). N, no treatment; D, Dex alone treatment; IC, injury and cytokine treatment; ICD, injury, cytokines, and Dex. Percentages on axes represent percent variance explained by that principal component

MS analysis identified 18,913 peptides with 2041 identified and quantified proteins in the media, and 8732 peptides corresponding to 1389 proteins in the cartilage tissue. The raw data are available via ProteomeXchange with identifier PXD032213 [32]. After filtering as described in the methods, the media contained 8718 peptides corresponding to 1451 proteins, and the cartilage tissue 7082 peptides and 1141 proteins. Filtered imputed data for individual peptides and proteins can be found in Supplemental File 1. The biological processes with the greatest representation in proteins identified in the media were cellular process, metabolic process, and biological regulation (Supplemental Figure 4A), with the same processes represented in the tissue proteome as well (Supplemental Figure 4B). PCA revealed that injury and cytokine treatment had the strongest contribution to sample variability in the media, while the donor effect was more significant for tissue samples (Fig. 2).

IC treatment caused an increase in the release of extracellular matrix (ECM) components (e.g., COMP, collagens III, V, VI, lumican), proteases (e.g., MMPs −1, −2, −3, −9, −10, −13, −14), pro-inflammatory factors (e.g., lipopolysaccharide-binding protein, serum amyloid A1, complement subcomponents 1R and 1S), and intracellular proteins (e.g., many ribosomal proteins, fructose-bisphosphate aldolase A, heat shock proteins) to the media (Table 1, Supplemental File 2). The major effect on the tissue proteome was a loss of intracellular metabolic proteins, and an increase in MMP2, MMP3, SOD2, and collagen II. Adding Dex to IC-treated osteochondral plugs reduced the media release of ECM proteins including aggrecan, collagen VII, lumican, and fibrillin-2, as well as proteases including MMPs −1, −3, −9, and −10. Many pro-inflammatory factors including interleukin-11, CXCL1, and complement C2, had increased levels with ICD treatment compared to IC, while in the tissue there was no effect on protein abundances. Dex alone reduced the release of some collagens (I, II, IX, XI, XII) and MMPs (−1, −13, −14) into the media, with mixed effects on protease inhibitors. Dex had little effect on the tissue proteome normalized to DNA content (Table 2), with a notable increase in tissue levels of matrix metalloproteinase 3 (MMP3) and superoxide dismutase 2 (SOD2), and a decrease in tissue levels of procollagen-lysine, 2-oxoglutarate 5-dioxygenase 1 and 2 (PLOD1 and 2), and collagen XI. Proteins and peptides found in only one or two treatment conditions, classified as those with quantified abundances in at least six of the seven donors for media samples or all four donors for tissue samples are listed in Supplemental File 3. Statistical results for both DNA normalized and non-DNA normalized cartilage tissue data are included in Supplemental Files 4 and 5, respectively.

**Table 1:** Biological process and molecular function enrichment for media proteins significantly affected by Dex/injury treatment. Proteins with a significant effect (either increased or decreased, *p* < 0.05) of Dex versus control (D/N), mechanical injury and cytokine treatment versus control (IC/N), or injury, cytokines, and Dex versus injury and cytokines (ICD/IC) were analyzed with STRING protein association network analysis, and the five biological processes and molecular functions with the strongest enrichment (log_10_(number of observed proteins/number of expected proteins)) were selected

	Biological process	FDR	Molecular function	FDR
**D/N**				
Increased	Cellular response to vitamin k	0.0282	Fibronectin binding	0.00012
	Negative regulation of smooth muscle cell-matrix adhesion	0.0282	Proteoglycan binding	2.03E−06
	UDP-glucuronate biosynthetic process	0.0418	Collagen binding	1.08E−06
	Positive regulation of substrate-dependent cell migration, cell attachment to substrate	0.0418	Laminin binding	0.04
	Negative regulation of plasminogen activation	0.005	Lipoprotein particle binding	0.04
Decreased	Positive regulation of cell proliferation by vegf-activated pdgf receptor signaling pathway	0.0029	Procollagen-lysine 5-dioxygenase activity	0.0066
	Esophagus smooth muscle contraction	0.0383	Phosphodiesterase i activity	0.0213
	Hydroxylysine biosynthetic process	0.0383	Platelet-derived growth factor binding	0.00028
	Glomerular capillary formation	0.0093	Extracellular matrix structural constituent conferring tensile strength	2.89E−10
	Peptidyl-lysine hydroxylation	0.0153	Low-density lipoprotein particle binding	0.0159
**IC/N**				
Increased	Actin filament fragmentation	0.0023	Peroxiredoxin activity	0.00033
	Helper t cell extravasation	0.0151	Thioredoxin peroxidase activity	0.0108
	Cellular hyperosmotic salinity response	0.0151	Copper chaperone activity	0.0108
	Positive regulation of establishment of protein localization to telomere	2.99E−06	Extracellular matrix constituent conferring elasticity	0.0027
	CRD-mediated mRNA stabilization	0.0037	Threonine-type endopeptidase activity	1.35E−06
Decreased	Collagen fibril organization	4.85E−05	Extracellular matrix structural constituent conferring tensile strength	1.31E−07
	Chondroitin sulfate proteoglycan biosynthetic process	0.0053	Heparan sulfate proteoglycan binding	0.0126
	Chondroitin sulfate biosynthetic process	0.0275	Insulin-like growth factor binding	0.00021
	Protein hydroxylation	0.0301	Extracellular matrix structural constituent	3.48E−09
	Chondroitin sulfate proteoglycan metabolic process	0.0025	Proteoglycan binding	0.0131
**ICD/N**				
Increased	Actin filament fragmentation	0.0031	Peroxiredoxin activity	0.00041
	Fumarate metabolic process	0.0188	Threonine-type endopeptidase activity	5.16E−12
	Helper t cell extravasation	0.0188	Thioredoxin peroxidase activity	0.0143
	Protein unfolding	0.0188	Copper chaperone activity	0.0143
	Cellular hyperosmotic salinity response	0.0188	Extracellular matrix constituent conferring elasticity	0.004
Decreased	Hydroxylysine biosynthetic process	0.0346	Procollagen-lysine 5-dioxygenase activity	0.0021
	Peptidyl-lysine hydroxylation	0.0112	Insulin-like growth factor ii binding	0.0145
	pdgf receptor-beta signaling pathway	0.0112	Extracellular matrix structural constituent conferring tensile strength	3.20E−08
	Type b pancreatic cell proliferation	0.0137	Insulin-like growth factor i binding	0.0242
	Basement membrane assembly	0.0137	Platelet-derived growth factor binding	0.0291
**ICD/IC**				
Increased	Negative regulation of plasminogen activation	0.0011	Threonine-type endopeptidase activity	1.11E−07
	Viral translational termination-reinitiation	0.0126	Peptide disulfide oxidoreductase activity	0.0031
	Proteasomal ubiquitin-independent protein catabolic process	4.16E−08	S100 protein binding	0.0041
	Modulation of age-related behavioral decline	0.0033	Fibroblast growth factor binding	0.0136
	Negative regulation of dendritic cell apoptotic process	0.0224	Low-density lipoprotein particle receptor binding	0.0153
Decreased	Hydroxylysine biosynthetic process	0.0235	Heparan sulfate proteoglycan binding	0.0021
	Gonadotrophin-releasing hormone neuronal migration to the hypothalamus	0.0345	CXCR chemokine receptor binding	0.0478
	Positive regulation of cell proliferation by vegf-activated platelet-derived growth factor receptor signaling pathway	0.0345	Fibronectin binding	0.0088
	Formaldehyde catabolic process	0.0345	Extracellular matrix structural constituent conferring tensile strength	0.0096
	Facioacoustic ganglion development	0.0345	Laminin binding	0.0105

*FDR*: False discovery rate

**Table 2:** Biological process and molecular function enrichment for tissue proteins significantly affected by Dex/injury treatment. Proteins with a significant effect (either increased or decreased, *p* < 0.05) of mechanical injury and cytokine treatment versus control (IC/N), or injury, cytokines, and Dex versus injury and cytokines (ICD/IC) were analyzed with STRING protein association network analysis, and the five biological processes and molecular functions with the strongest enrichment (log_10_(number of observed proteins/number of expected proteins)) were selected

	Biological process	FDR	Molecular function	FDR
**IC/N**				
Increased	Negative regulation of plasminogen activation	0.0409	Protease binding	0.0064
	Negative regulation of metallopeptidase activity	0.049	Endopeptidase inhibitor activity	0.0182
	Extracellular matrix disassembly	4.33E−06	Enzyme inhibitor activity	0.0483
	Collagen catabolic process	0.0033	Signaling receptor binding	7.85E−05
	Regulation of cellular senescence	0.0495	Binding	0.0182
Decreased	Hydroxylysine biosynthetic process	0.0491	Procollagen-lysine 5-dioxygenase activity	0.0042
	Isocitrate metabolic process	0.0092	Procollagen-proline dioxygenase activity	0.0164
	Valine metabolic process	0.0092	Peptide disulfide oxidoreductase activity	0.0042
	Peptidyl-lysine hydroxylation	0.016	Racemase and epimerase activity, acting on carbohydrates and derivatives	0.0404
	Positive regulation of rna polymerase ii transcription preinitiation complex assembly	0.0252	L-ascorbic acid binding	0.0011
**ICD/N**				
Decreased	Telomerase holoenzyme complex assembly	0.0074	Procollagen-lysine 5-dioxygenase activity	0.0075
	Isocitrate metabolic process	0.00088	Proteasome-activating atpase activity	0.0075
	Positive regulation of rna polymerase ii transcription preinitiation complex assembly	0.00022	Isocitrate dehydrogenase activity	0.0168
	Valine metabolic process	0.0153	Procollagen-proline dioxygenase activity	0.0313
	Positive regulation of establishment of protein localization to telomere	0.0032	Peptide disulfide oxidoreductase activity	0.001

*FDR*: False discovery rate

### Disease and Dex effects on cartilage tissue and bone homeostasis

**Fig. 3:**
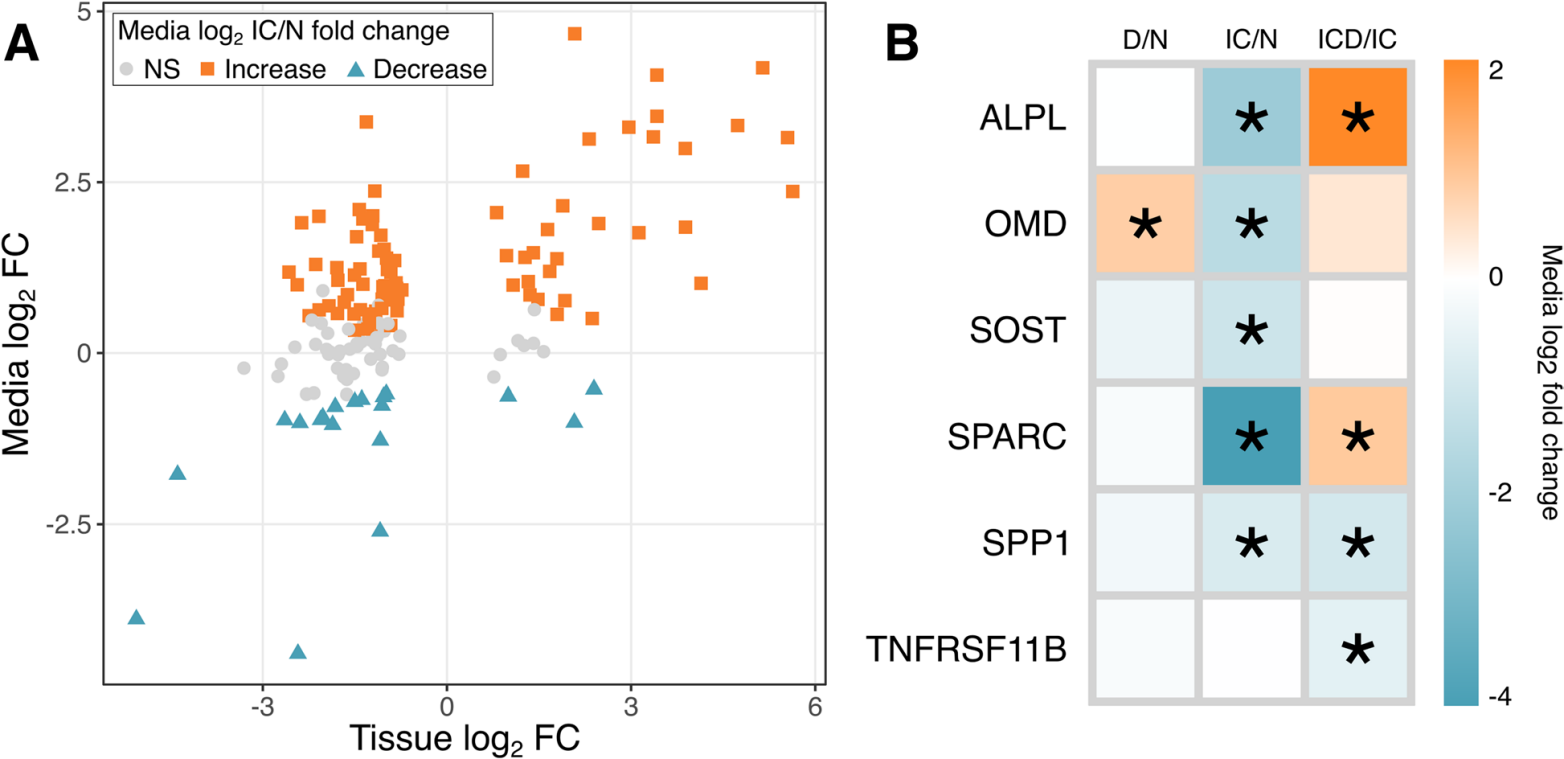
Effects of disease on cartilage tissue and biomarkers of bone homeostasis. **A**. To compare changes in the tissue proteome with IC treatment to the behavior of those same proteins in the media, the log_2_ fold changes (FC) of proteins with a significant effect with injury and cytokine (IC) treatment versus control (N) in cartilage tissue were plotted against their media log_2_ FC of IC/N. Colors represent significance and direction of effect in media. **B**. Log_2_ fold change of media levels for selected biomarkers of bone health across all treatment conditions. N, no treatment; D, Dex alone treatment; IC, injury and cytokine treatment; ICD, injury, cytokines, and Dex; ALPL, alkaline phosphatase; OMD, osteomodulin; SPARC, osteonectin; SPPI, osteopontin; SOST, sclerostin; TNFRSF11B, osteoprotegerin. *: *p* < 0.05

To investigate proteins identified in both the tissue and media proteomes and their behavior in each compartment under disease stress, we compared the proteins with a significant effect of IC treatment in the tissue to their corresponding changes in the media with IC treatment (Fig. 3A). Some proteins had a decrease in both media and tissue, including PLOD1, collagen IX, and collagen XI. The majority had a decrease in the tissue and a corresponding increase in the media, which had many intracellular metabolic and homeostatic proteins such as ribosomal proteins, alcohol dehydrogenase, and protein disulfide-isomerase A6. Proteins with increased levels in both tissue and the media included MMPs −1, −2, and −3, serpin family E member 1 and member 2 (SERPINE1 and -2), and SOD2.

Media levels of osteomodulin (OMD), osteopontin (SPP1), osteonectin (SPARC), sclerostin (SOST), and alkaline phosphatase (ALPL) were decreased with IC treatment up to 16-fold (Fig. 3B). With ICD treatment, media levels of SPARC and ALPL were increased compared to IC treatment, but SPP1 was further decreased. Dex alone only caused the increase of OMD abundance in the media. Osteoprotegerin (TNFRSF11B) did not experience a change with Dex or IC treatment alone and was slightly decreased by Dex addition to IC treatment compared to IC only.

### Analysis of matrix breakdown at the peptide level

**Fig. 4:**
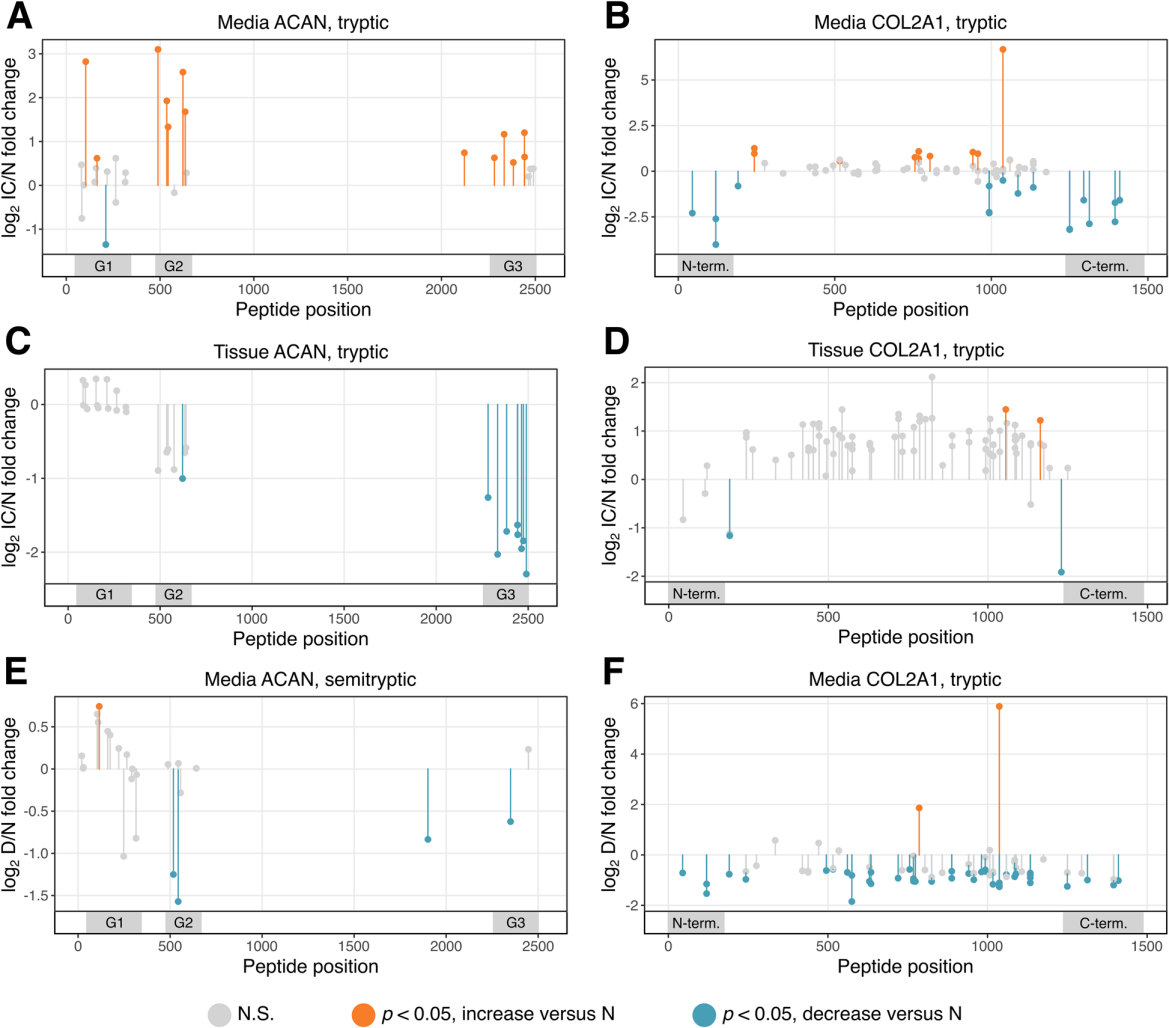
Effects of disease and Dex on individual tryptic and semitryptic peptides from aggrecan and collagen II. Log_2_ fold change of injury and cytokine treatment versus control (**A**–**D**) or Dex versus control (**E**, **F**) abundances of peptides identified in aggrecan (ACAN; **A**, **C**, **E**) and collagen II (COL2A1l; **B**, **D**, **E**). Peptides identified in media (**A**, **B**, **E**, **F**) or tissue (**C**, **D**). x-axis: residue position of first amino acid for each peptide. Orange: significant (*p* < 0.05) increase compared to control. Blue: significant (*p* < 0.05) decrease compared to control. Gray: no significant change compared to control (N.S.) Positions of globular domains for aggrecan (G1, G2, G3) and collagen II N- and C-terminal regions (N-term. and C-term., respectively) are highlighted

Statistical results for individual peptides can be found in Supplemental Files 6 and 7 for media and non-DNA normalized cartilage tissue, respectively. Aggrecan (ACAN, Fig. 4A) had peptides identified in the G1, G2, and G3 globular domains that were mostly increased with IC treatment compared to control in the media. The tryptic peptides identified in the media for collagen II (COL2A1, Fig. 4B) were decreased with IC treatment in the N- and C- terminal regions 2- to 5-fold but increased in the center of the protein. In the tissue, all tryptic peptides identified in the G3 domain of aggrecan had decreased release compared to control under injury and disease stress (Fig. 4C), and the effect on collagen II was only significant in the C-terminal region (Fig. 4D). Semitryptic peptides found only in the IC-treated condition were found from proteins including fibronectin-1 (FN1), COMP, biglycan (BGN), and collagen VI in the tissue, and collagen II, MMP3, MMP13, fibrillin-1 (FBN1), and FN1 in the media (Supplemental File 3).

Dex had mixed effects on semitryptic peptides, those generated by endogenous proteases and not through trypsin digestion during MS preparation. Many proteins with several identified semitryptic peptides had opposite effects, such as aggrecan (Fig. 4E), where semitryptic peptides found in the G1 domain had increased release into the media with Dex treatment, but semitryptic peptides from the G2 and G3 domains were decreased compared to control. Dex broadly caused a decrease in media levels of peptides from collagen II (Fig. 4F), including in the N- and C-terminal regions, with only three having increased abundances compared to control. Collagen I (COL1A1) peptides in both the N- and C-terminal propeptide regions were decreased in the media with both Dex and IC treatment (Supplemental Figure 5).

**Fig. 5:**
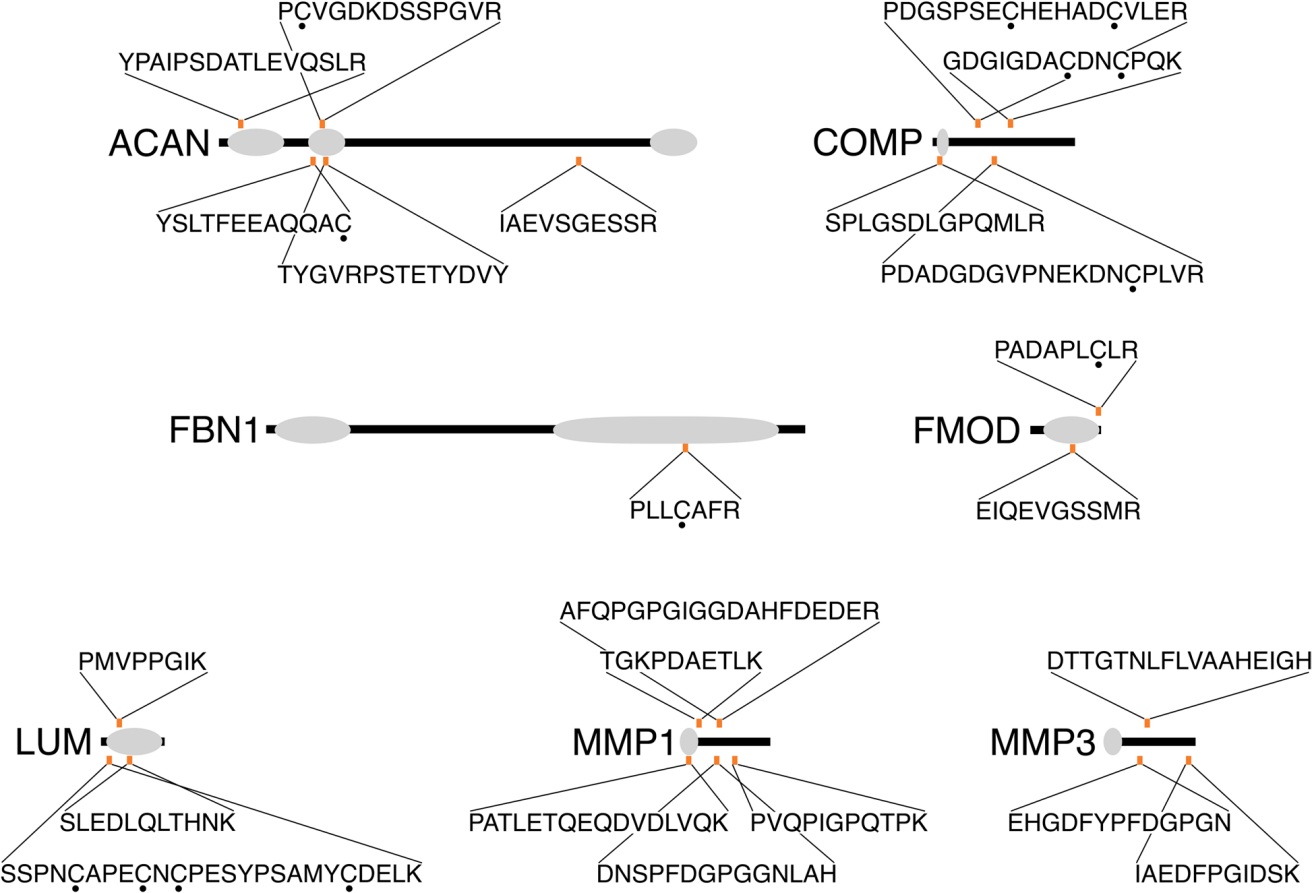
Semitryptic peptides associated with increased sGAG loss. Semitryptic peptide abundance data across all media samples were regressed against sGAG loss for each donor and treatment condition. Selected peptides from among the top 50 highest associated peptides are displayed on their corresponding position in their master protein. Gray areas represent notable domains for each protein; ACAN: globular domains; COMP: N-terminal domain, FBN1: N- and C-terminal domains; FMOD: leucine-rich repeats; LUM: leucine-rich repeats; MMP1: propeptide; MMP3: propeptide. • : Carbamidomethylation of cysteine residue

To investigate which semitryptic peptides may be biomarkers of disease progression, the abundances of each semitryptic peptide were averaged across three biological replicates within each treatment condition and donor, and then regressed against the total percent sGAG loss, an analog for disease severity, for that treatment condition and donor. From the top 50 peptides with the greatest association with increased sGAG loss, the most represented proteins were MMP1, ACAN, lumican (LUM), COMP, and MMP3, with other ECM components fibromodulin (FMOD), and FBN1 notably among the top 50 as well (Fig. 5).

### Donor-specific effects on sGAG loss and media proteome

**Fig. 6:**
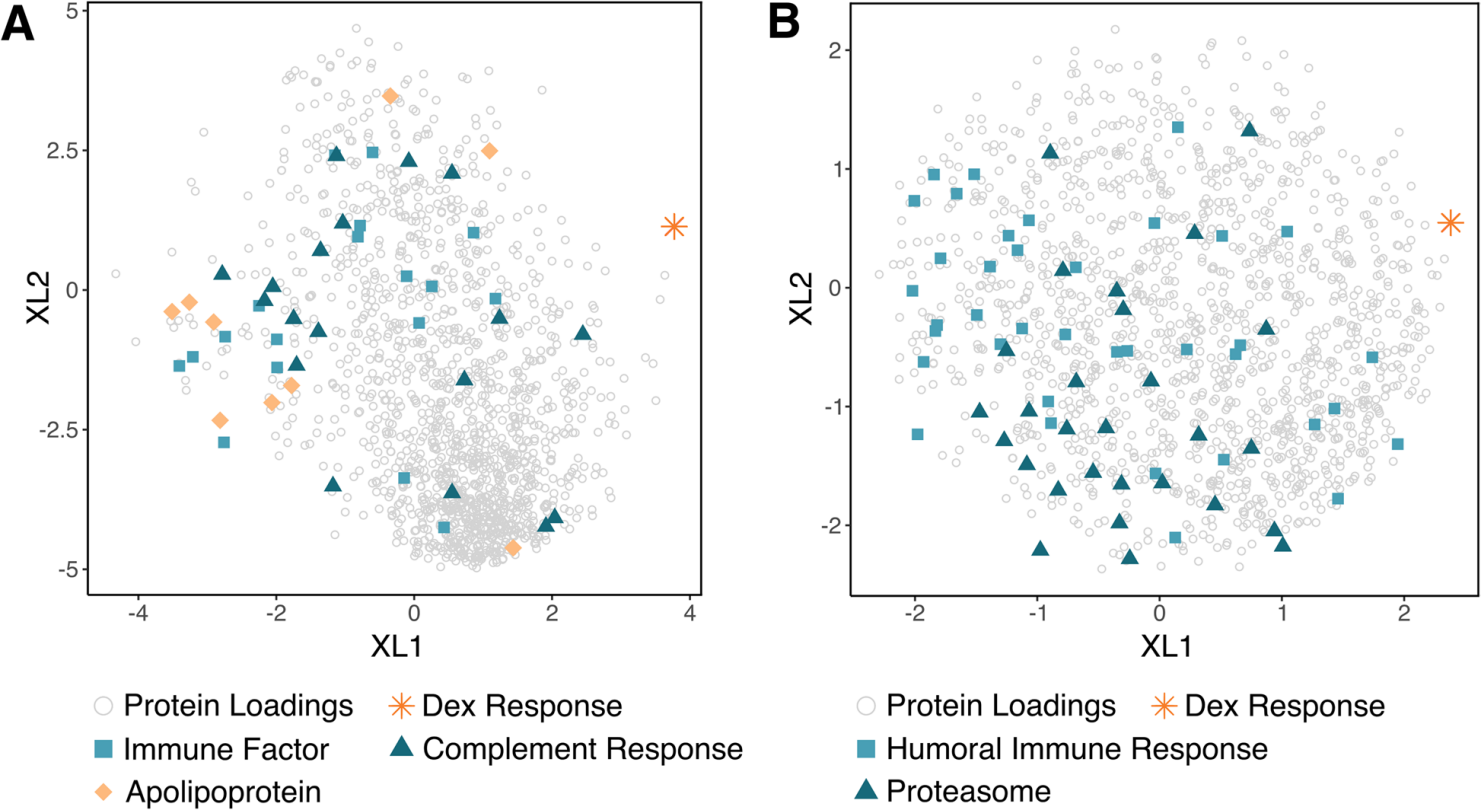
Partial least squares regression of Dex attenuation of sGAG loss. **A**. Protein loadings after PLSR of all batch-corrected media proteomic data against the percent of increased sGAG loss caused by IC treatment that was reduced by Dex for each donor. Enriched biological processes were determined with Gene Set Enrichment Analysis. XL: predictor loading values. **B**. PLSR of only untreated control media against the percent Dex attenuation of increased sGAG loss with IC treatment

To investigate what protein biomarkers in the media correlated with Dex response, we calculated the percent of the increased sGAG loss caused by IC treatment compared to control that was attenuated by Dex for each donor, and regressed the batch-corrected media proteomic data against this percent reduction. When regressing all samples, apolipoproteins, proteins involved in the complement response, and pro-inflammatory factors were all associated with a lower response to Dex, i.e. less of an attenuation of increased sGAG loss with IC treatment (Fig. 6A). To look for potentially predictive markers of a lack of a Dex response, we regressed only the control samples against the Dex percent reduction (Fig. 6B). As with the regression against all media samples, higher levels of proteins associated with humoral immune responses was associated with a lack of a Dex response, as well as proteasomes 20S and 26S.

## Discussion

In this ex vivo model of human ankle PTOA, we sought to answer how initial stages of cartilage catabolism and anabolism occur in the presence of both cartilage and underlying bone, and what donor-specific responses to both disease and drug treatment would occur. This system used healthy primary osteochondral plugs, rather than total joint replacement discards, as is often the default for in vitro osteochondral studies [5]. IC treatment increased the release of ECM components, proteases, pro-inflammatory factors, and intracellular proteins to the media, with mixed effect on protease inhibitors. This agrees with previous models using isolated human knee cartilage [9, 15]. The addition of Dex to injury and cytokine-treated osteochondral explants reduced the release of many ECM proteins and proteases, consistent with previous conclusions on the anti-catabolic actions of Dex in models of PTOA [9, 12, 14, 15]. Many intracellular proteins identified in both the tissue and media proteomes had decreased levels in tissue and corresponding increases in the media under injury and cytokine stress, and under IC stress, there was a significant amount of chondrocyte death compared to control. As previously hypothesized, the presence of these proteins in the media could be markers of early necrotic cell death [9, 15].

This model allowed for the analysis of specific mechanisms of matrix breakdown by observing the behavior of individual regions of proteins such as aggrecan. Under disease stress, the G2 and G3 globular domains saw significant levels of cleavage and release into the media. The aggrecan found in the tissue was depleted for peptides from the G3 region, which supports previous findings that aggrecan is degraded primarily from the G3 domain in the N-terminal direction towards the other interglobular regions [11, 33, 34]. Decreased abundances of N- and C- terminal domain peptides in the tissue and media were also observed: the N- and C- terminal domains of collagen II are cleaved during maturation and incorporation into the matrix, and lower levels of these peptides in the media and tissue suggest decreases in the synthesis of new collagen II and suppression of anabolic repair [35]. Dex also suppressed the synthesis of new collagen II as indicated by lower levels of the N- and C-terminal peptides in the media. Additionally, Dex affected the activity of proteases and protease inhibitors in healthy tissue, as based on increases in the release of specific semitryptic peptides from aggrecan and decreasing the release of others.

Bone also undergoes changes due to disease and Dex treatment. OMD, SPP1, SPARC, SOST, and ALPL are common biomarkers of bone health and disease responses. All were decreased in the media with IC treatment compared to controls. Some of the selected biomarkers could be protective under osteoarthritic stress: OMD stimulates osteogenesis, and SOST was protective of cartilage viability in a mouse model of OA/PTOA [36–38]. The synthesis of collagen I, an indicator of osteoblast metabolism, was decreased under disease stress based on levels of its propeptides in the media [39]. The results presented here contradict previous reports on the presence of these biomarkers under OA stress. SPP1, ALPL, and SPARC have all been shown to be increased in models of OA or associated with OA severity in patient serum [40–42]. This could be due to the lack of synovial and other joint tissues or the shorter time span of this model compared to long-term PTOA progression [40, 43]. Corticosteroids are also expected to affect bone biology, as at certain doses and durations are considered detrimental to bone health [18, 44]. However, Dex rescued decreased levels of ALPL and SPARC when added to IC-treated osteochondral explants, but further decreased the levels of SPP1 and collagen I synthesis. Dex alone only caused a change in secreted OMD, increasing the amount of this osteogenic biomarker. The relatively short duration (3 weeks) and low dose of Dex used in our study (100 nM) may mitigate against strong catabolic effects on the bone.

As semitryptic peptides are generated by endogenous protease activity and not by trypsin treatment during MS preparation, these peptides can serve as potential biomarkers for the identification of PTOA progression [10]. Proteases including MMPs are synthesized and released at the earliest stages of PTOA progression, so semitryptic peptides are promising candidates for prognostic PTOA biomarkers [9]. Regression of semitryptic peptides identified in this study against sGAG loss identified semitryptic peptides from aggrecan, COMP, FBN1, FMOD, LUM, MMP1, and MMP3 as those most predictive of disease severity. These are promising candidates for PTOA biomarkers that should be investigated further in clinical samples to determine their potential prognostic use.

Biomarkers are also desirable to determine which patients might respond best to a therapeutic such as Dex [45, 46]. Proteomic data were regressed against the amount of sGAG lost under disease stress that was attenuated by Dex treatment for each donor in order to determine which proteins best predicted anti-catabolic activity. Humoral immune proteins and the pro-inflammatory apolipoproteins A, B, C, and H were all inversely associated with a Dex attenuation of sGAG loss, while the anti-inflammatory apolipoprotein E was associated with a Dex attenuation effect [47–49]. However, apolipoproteins A and B have also been shown to have anti-inflammatory activity, inhibiting T cell activation and the innate immune response [50]. When regressing only the untreated control condition, most analogous to healthy patients or very early stages of the disease, a lack of a Dex attenuation response was also associated with proteasomes 20S and 26S. Proteasomes play a role in T cell activation and immune responses, and circulating proteasomes have been associated with immunological activity and cellular damage [51, 52]. These results suggest that a highly elevated initial inflammatory state could prevent the anti-catabolic activity of Dex, which may help to distinguish patients that may best respond to Dex. Variability in donor responses to disease as well as the baseline inflammatory state of a joint are likely tied to sex, diet, BMI, lifestyle, genetic disposition, cartilage thickness, and many other factors that cannot be explored with such a small number of donors [53]. Further investigation of clinical samples from many Dex-treated patients is necessary to follow up on these results.

### Study limitations

PTOA of the talocrural joint is a relevant model system to study due to the high incidence of ankle PTOA, but these results must be validated in samples from knee and hip cartilage to confirm their translatability to those joints that may have different treatment and disease responses [22, 54]. Cartilage thickness of each osteochondral plug varied within and between donors, though normalizing to cartilage weight and DNA content addressed differences in both the amount of tissue and number of cells within the tissue. Osteochondral plugs were randomized between treatment conditions to further address location-specific variation. Osteochondral plugs had differences in bone density, vascularity, and cellularity, and while the bone could be cut to approximately the same length, it did not always cut perfectly. Our present results confirm several of those previous findings of disease and Dex effects [15], but notably used a single concentration of and continuous exposure to Dex, and these results must be explored in further experiments that compare different timings and doses of Dex treatment. The current approach to intra-articular injection of corticosteroids involves a much higher initial dose that is maintained for a much shorter time due to rapid clearance from the joint. However, novel methods of drug delivery are currently under investigation to replicate low-dose Dex delivery over a longer durations targeted to cartilage [55–57]. The addition of synovial tissue to this system would broaden applicability to knee and other joints, but would introduce uncontrolled and inconsistent levels of inflammatory cytokines compared to controlled addition of exogenous cytokines [58]. Our proteomic analysis used MS identification based on trypsin digestion of media and tissue proteins, which removes the ability to identify some endogenously produced peptides. We utilized a high threshold of confidence for identification and strict filtering for inclusion to compensate for any single-peptide identifications or quantifications. The tissue proteome only includes proteins that could be solubilized and extracted from the tissue. Proteins identified in the media by MS could have been released from both cartilage and bone, so it is difficult to explicitly say what tissue changes in media levels of proteins originate from.

## Conclusions

This osteochondral human ex vivo model of PTOA allowed for the analysis of disease progression and Dex effects in the context of both cartilage and bone starting with healthy tissue. The combination of a single mechanical injury, TNF-α, and IL-6 treatment caused catabolic changes and a suppression of cartilage anabolism, in line with other studies using isolated cartilage. This model of early PTOA caused some changes to bone metabolism as well, which did not extend to full bone breakdown but suppressed normal homeostatic processes. The addition of Dex to this PTOA model confirmed the anti-catabolic effects seen in previous studies using isolated cartilage, and peptidomic analyses revealed a suppression of collagen II synthesis and changes to endogenous protease activity, even with healthy cartilage. Elevated levels of humoral proteins and apolipoproteins were associated with a lack of a Dex response, a promising set of predictive biomarkers for patient Dex response that could allow for targeted, personalized drug treatment that can be combined with pro-anabolic drugs to offset potential off-target effects [14]. With Dex as a promising DMOAD candidate for early PTOA intervention, prognostic biomarkers are critical to determine patients most at risk of developing PTOA as well as those most likely to respond to Dex. Semitryptic peptide biomarkers associated with PTOA progression from several ECM proteins and proteases presented in this study should be validated against longitudinal clinical samples and used in tandem with other biomarker candidates, as with patient heterogeneity there is likely not only one single sufficient biomarker.

## Supplementary Information

**Additional file 1: Figure S1.** Donor differences in DNA content and batch effect in media proteome. **Figure S2.** Fluorescent imaging assessment of cartilage viability. **Figure S3.** GAG loss within individual donors. **Figure S4.** Proteomic identification of media and extracted tissue proteins. **Figure S5.** Changes in media abundances of collagen I tryptic peptides with Dex and disease treatment.

**Additional file 2.**

**Additional file 3.**

**Additional file 4.**

**Additional file 5.**

**Additional file 6.**

**Additional file 7.**

**Additional file 8.**

## Data Availability

The mass spectrometry proteomics data have been deposited to the ProteomeXchange Consortium (http://proteomecentral.proteomexchange.org) via the PRIDE partner repository [32] with the dataset identifier PXD032213.

## Abbreviations

ACAN: Aggrecan
ALPL: Alkaline phosphatase
BGN: Biglycan
COMP: Cartilage oligomeric matrix protein
D: 100 nM dexamethasone treatment
Dex: Dexamethasone
DMEM: Dulbecco’s modified Eagle’s medium
DMMB: Dimethylmethylene blue
DMOAD: Disease-modifying osteoarthritis drug
ECM: Extracellular matrix
FBN1: Fibrillin-1
FC: Fold change
FDR: False discovery rate
FMOD: Fibromodulin
FN1: Fibronectin-1
IC: Injury and cytokine treatment
ICD: Injury, cytokine, and 100 nM Dex treatment
IL-6: Interleukin-6
MMP: Matrix metalloproteinase
MS: Mass spectrometry
N: No treatment
OA: Osteoarthritis
OMD: Osteomodulin
PLOD: Procollagen-lysine, 2-oxoglutarate 5-dioxygenase
PTOA: Post-traumatic osteoarthritis
SERPINE: Serpin family E member
sGAG: Sulfated glycosaminoglycan
sIL-6R: Soluble IL-6 receptor
SOD2: Superoxide dismutase 2
SOST: Sclerostin
SPARC: Osteonectin
SPP1: Osteopontin
TNF-α: Tumor necrosis factor alpha
TNFRSF11B: Osteoprotegerin
