# Supplementary figures and images for: Tissue catabolism and donor-specific dexamethasone response in a human osteochondral model of post-traumatic osteoarthritis
Source: Arthritis Res Ther. 2022 Jun 10;24:137. doi: 10.1186/s13075-022-02828-4 (PMC9185927; doi:10.1186/s13075-022-02828-4)

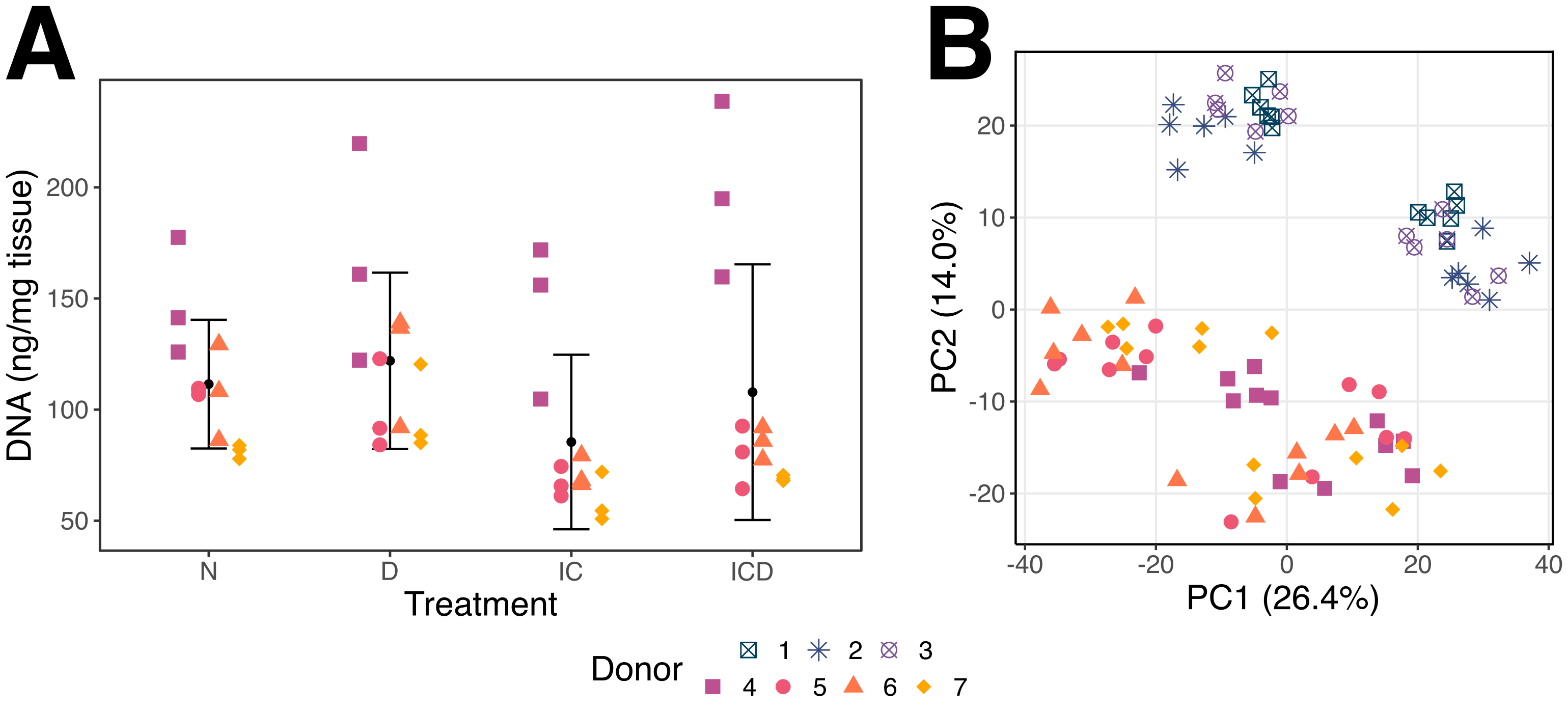

Supplement: Supplementary file 1 — Additional file 1: Figure S1. Donor differences in DNA content and batch effect in media proteome. Figure S2. Fluorescent imaging assessment of cartilage viability. Figure S3. GAG loss within individual donors. Figure S4. Proteomic identification of media and extracted tissue proteins. Figure S5. Changes in media abundances of collagen I tryptic peptides with Dex and disease treatment. [file 13075_2022_2828_MOESM1_ESM.zip › SFig1.tiff]

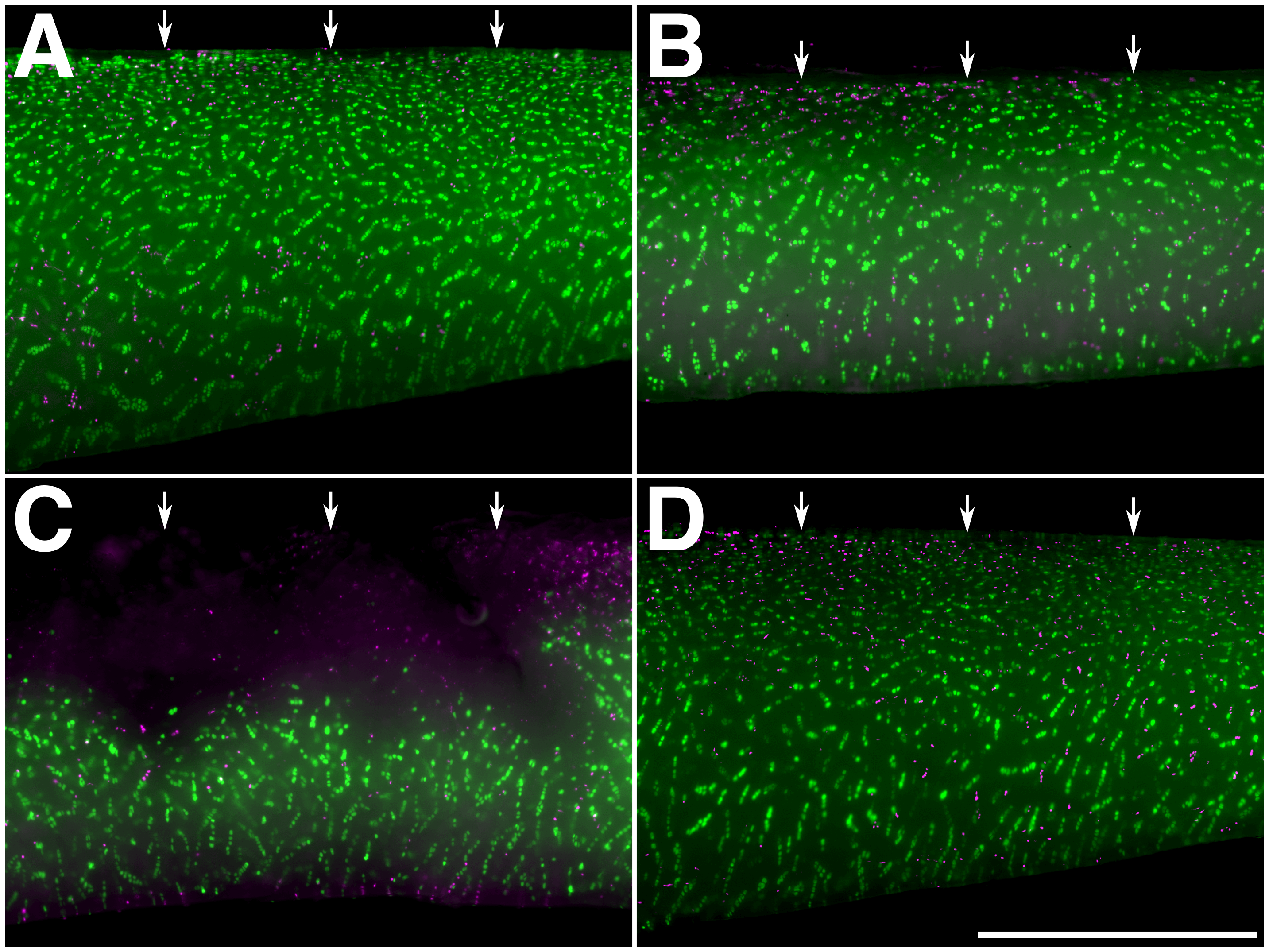

Supplement: Supplementary file 1 — Additional file 1: Figure S1. Donor differences in DNA content and batch effect in media proteome. Figure S2. Fluorescent imaging assessment of cartilage viability. Figure S3. GAG loss within individual donors. Figure S4. Proteomic identification of media and extracted tissue proteins. Figure S5. Changes in media abundances of collagen I tryptic peptides with Dex and disease treatment. [file 13075_2022_2828_MOESM1_ESM.zip › SFig2.tiff]

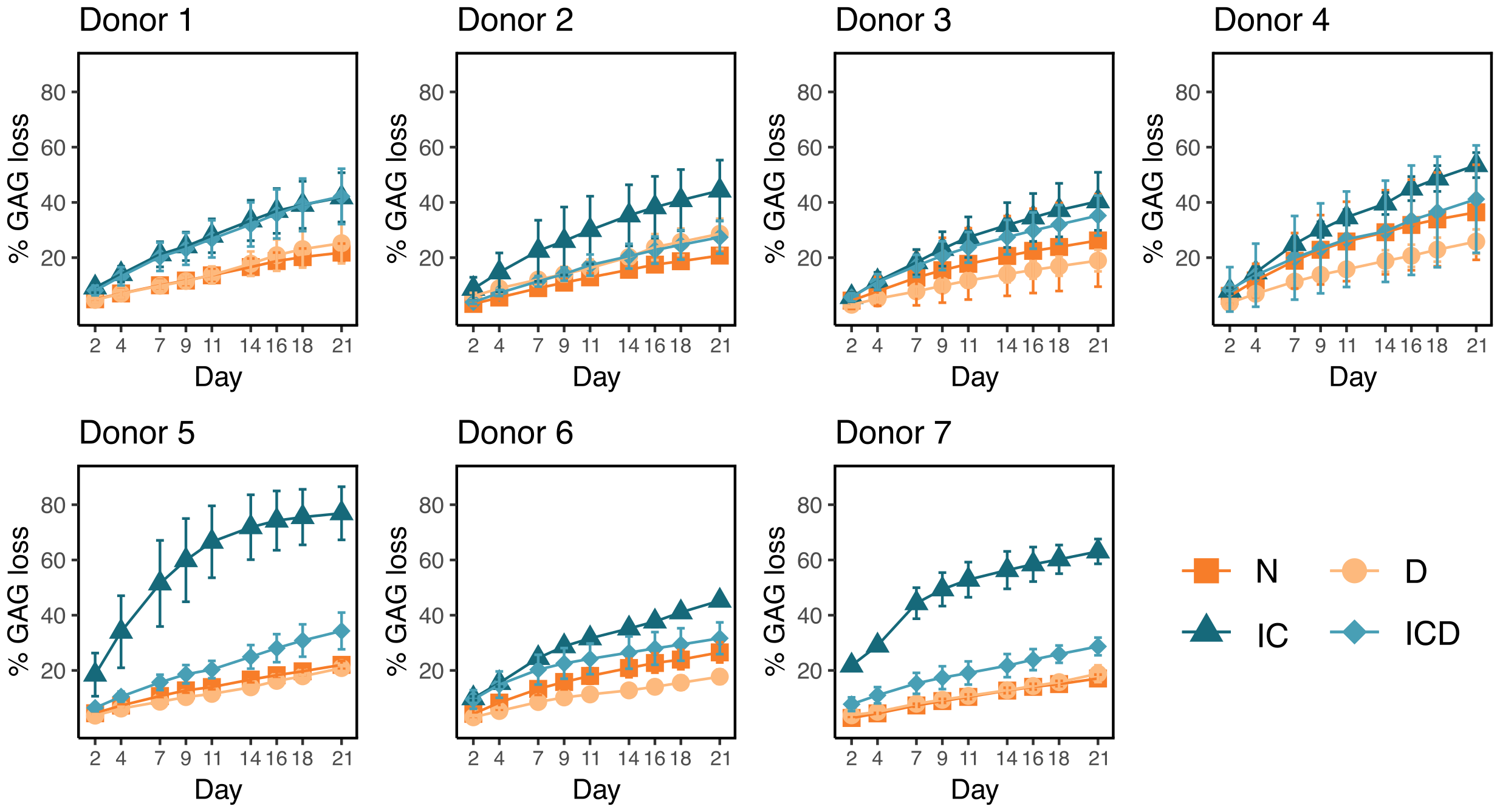

Supplement: Supplementary file 1 — Additional file 1: Figure S1. Donor differences in DNA content and batch effect in media proteome. Figure S2. Fluorescent imaging assessment of cartilage viability. Figure S3. GAG loss within individual donors. Figure S4. Proteomic identification of media and extracted tissue proteins. Figure S5. Changes in media abundances of collagen I tryptic peptides with Dex and disease treatment. [file 13075_2022_2828_MOESM1_ESM.zip › SFig3.tiff]

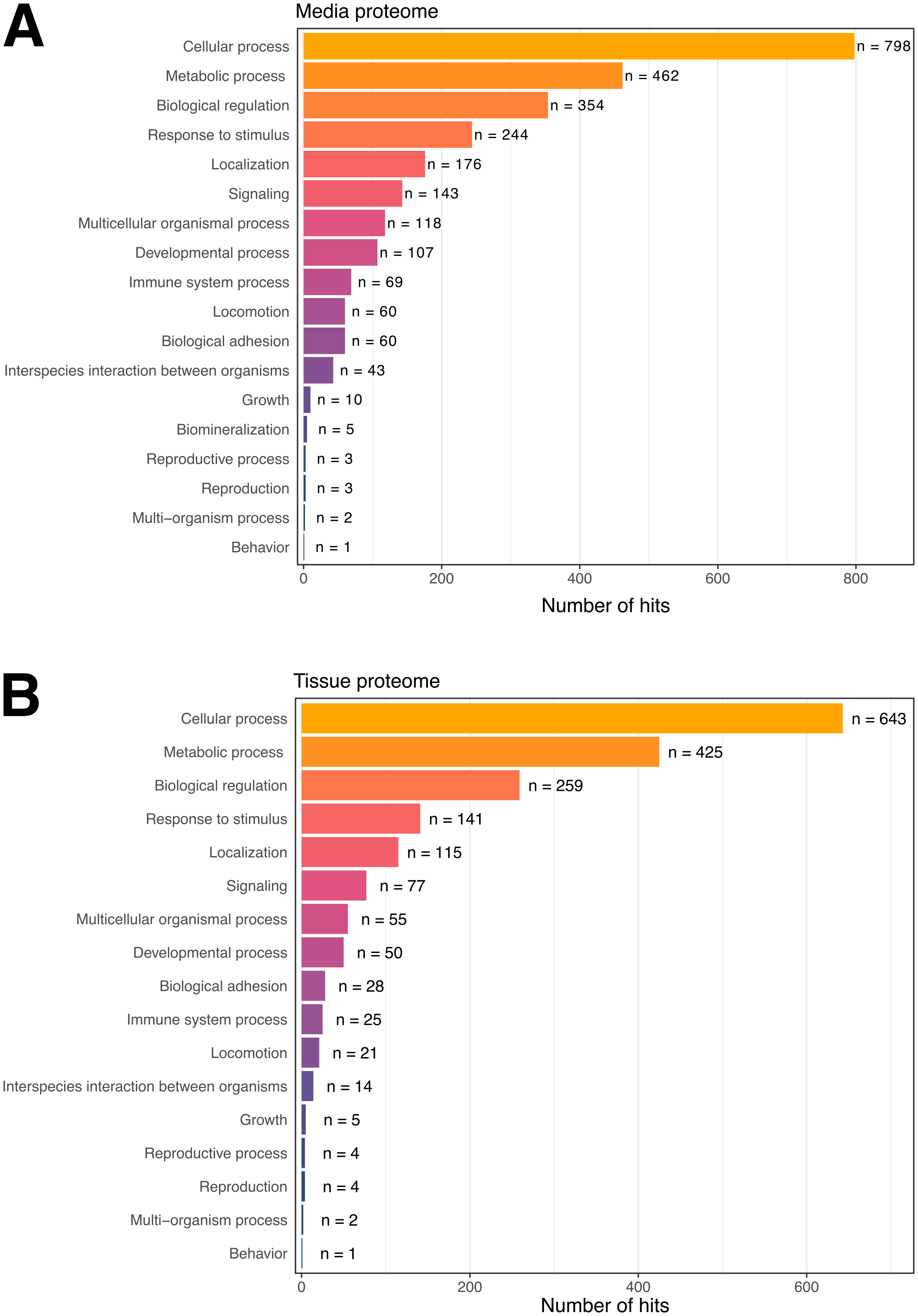

Supplement: Supplementary file 1 — Additional file 1: Figure S1. Donor differences in DNA content and batch effect in media proteome. Figure S2. Fluorescent imaging assessment of cartilage viability. Figure S3. GAG loss within individual donors. Figure S4. Proteomic identification of media and extracted tissue proteins. Figure S5. Changes in media abundances of collagen I tryptic peptides with Dex and disease treatment. [file 13075_2022_2828_MOESM1_ESM.zip › SFig4.tiff]

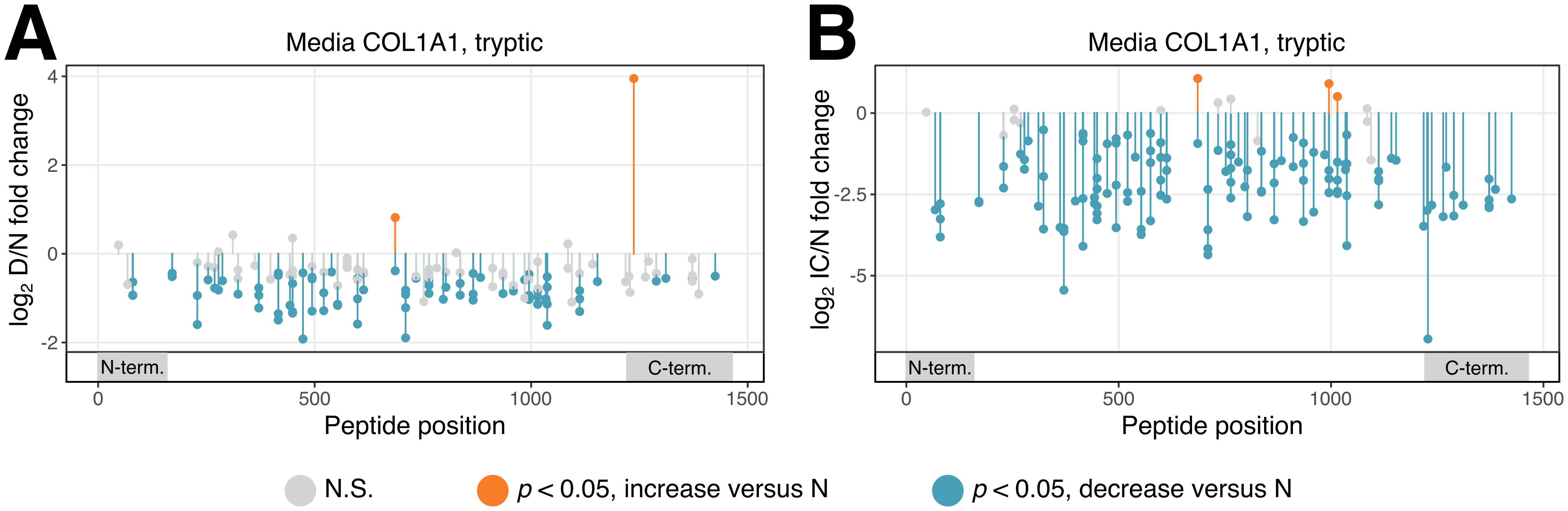

Supplement: Supplementary file 1 — Additional file 1: Figure S1. Donor differences in DNA content and batch effect in media proteome. Figure S2. Fluorescent imaging assessment of cartilage viability. Figure S3. GAG loss within individual donors. Figure S4. Proteomic identification of media and extracted tissue proteins. Figure S5. Changes in media abundances of collagen I tryptic peptides with Dex and disease treatment. [file 13075_2022_2828_MOESM1_ESM.zip › SFig5.tiff]
